# Synthesis, characterization and application of a non-flammable dicationic ionic liquid in lithium-ion battery as electrolyte additive

**DOI:** 10.1038/s41598-020-66341-x

**Published:** 2020-06-15

**Authors:** Kajari Chatterjee, Anil D. Pathak, Avinash Lakma, Chandra Shekhar Sharma, Kisor Kumar Sahu, Akhilesh Kumar Singh

**Affiliations:** 1 School of Minerals, Metallurgical and Materials Engineering, Indian Institute of Technology Bhubaneswar, Bhubaneswar, 752050 India; 2 School of Basic Sciences, Indian Institute of Technology Bhubaneswar, Bhubaneswar, 752050 India; 3 Creative & Advanced Research Based On Nanomaterials (CARBON) Laboratory, Department of Chemical Engineering, Indian Institute of Technology, Kandi, Hyderabad, 502285 Telangana India

**Keywords:** Batteries, Energy storage

## Abstract

A novel dicationic room temperature ionic liquid, 1,1′-(5,14-dioxo-4,6,13,15-tetraazaoctadecane-1,18-diyl) bis(3-(sec-butyl)-1H-imidazol-3-ium) bis((trifluoromethyl)-sulfonyl) imide has been synthesized and fully characterized. Its thermal and electrochemical analyses along with transport properties have been studied. We propose it as a potential nominal additive to the commonly used conventional organic carbonate electrolyte mixture and study its adaptability in Lithium-ion batteries which are the prime power sources for ultraportable electronic devices. We have compared the performance characteristics of the full cells made without and with this ionic liquid. The cells comprise lithium nickel cobalt manganese oxide cathode, graphite anode and ethylene carbonate - dimethyl carbonate (1:1, v/v + LiPF_6_) mixture electrolyte with nominal amount of ionic liquid as additive. The major concern with conventional electrolytes such as degradation of the materials inside batteries has been addressed by this electrolyte additive. Additionally, this additive is safer at relatively higher temperature. In its presence, the overall battery life is enhanced and it shows good cycling performance and coulombic efficiency with better discharge capacities (22% higher) after 100 cycles. Even after the increase in current rate from 10 mA/g to 100 mA/g, the cell still retains around 73% of capacity.

## Introduction

Lithium-ion batteries (LIBs)^1,2^ which possess high energy density have been in high demand as energy storage solution in lot of portable electronic gadgets/devices such as mobile phones and laptops. Further, they have the potential to serve as the most promising energy storage options for the next generation electric vehicles and smart grid technology^3,4^. The enhancement of operational safety in LIBs has been a major concern for last few decades. The stringent requirement for enhanced safety compels extensive studies those are focused towards the improvement in their safety and stability for long cycles without compromising on their performance^5^. The commercial batteries available in the market are mainly fabricated with organic carbonates along with lithium hexafluorophosphate (LiPF_6_) as electrolytes^6–8^, which are prone to ignition or even explosion when cells are damaged or exposed to high temperatures. Safety should be the core of the design philosophy of a battery^9–11^. In an excellent review, Zhang has meticulously discussed the effect of electrolyte additives to enhance the performance of LIBs by many fold like facilitating the formation of SEI, increase in cycling of LIB, enhancing thermal stability as compared to carbonate based organic electrolytes, as a cathode protecting material and even improving the physical properties of the electrolytes^10^. Han *et al*.^12^ and Kang *et al*.^13^ showed that additives can enhance the electrochemical cell performances of LIBs. During the cyclical operations of these cells, the instability of the Li/electrolyte interface might end up into short-circuiting of the cells due to dendrite formation on electrodes. To increase the safety and stability along with the performance of LIBs, lots of efforts are being made globally towards the development of more suitable electrolytes^14–21^. To be particular, Haregewoin *et al*.^15^ have discussed the different roles of electrolytes additives for LIBs while Kalhoff *et al*.^19^ have discussed the safety aspects of electrolytes for LIBs.

In this context, ionic liquids have off-late emerged as a new class of material and have attracted much attention for their potential applications as electrolytes^22–25^ in lithium ion batteries^26–28^, proton exchange membranes in fuel cells^29^, high energy density supercapacitors^30–32^ among others. Ionic liquids^33,34^ have emerged as a plausible solution for these issues because of their relatively superior thermal and electrochemical stability, flame retardant performance, tunable solvation properties and negligible vapour pressure therefore meeting most of the primary safety requirements. T.Yim *et al*.^35^ elaborately discussed the advantage of room temperature ionic liquid based electrolyte as an alternative to the conventional carbonate based electrolyte in LIBs. Due to the favourable physicochemical and electrochemical properties of the imidazolium-based ionic liquids, they are attractive candidates as non-volatile, non-flammable, and high voltage electrolyte materials for lithium ion batteries^28,36,37^ and are expected to enhance its safety performances considerably. However, the ionic liquid might not be appropriate candidates for standalone electrolytes for LIBs because of their high viscosity along with relatively low ionic conductivity^38^.Several studies proposed using ionic liquids as electrolyte additives in LIBs. Sato et. al. have shown that aliphatic quaternary ammonium salt containing carbonate solvents as electrolytes can be used to enhance the performance of lithium ion cells^39^. Zheng et. al. have shown that, addition of 20 vol% of organic carbonate solvents into the ionic liquid electrolyte prevents the intercalation of the organic cations and helps the formation of the graphite interlayer compound of lithium^40^. The properties of ionic liquids can be finely tuned to a large extent^41–43^ for their adaptation to a targeted use. In the context of application of ionic liquids in LIBs, mostly the monocationic ionic liquids with an anion have been explored. However, dicationic ionic liquids (DILs), a new class of ionic liquid, might be more suitable for such applications^44,45^ as they offer greater flexibility in tailoring to the desired variations of the cationic as well as anionic species. Most notably, DILs possess better thermal stability, wider liquid range and higher melting point^46–48^. Zhang *et al*.^45^ showed asymmetrical DIL, MIC*n*N_111_-TFSI_2_ can improve the cell performances than that of conventional electrolytes. Design options can be broadened to include several categories such as homoanionic, heteroanionic, symmetrical and asymmetrical DILs. Guided by these design clues, we engineered a novel urea functionalized imidazolium-based homoanionic symmetrical (geminal) dicationic ionic liquid (IL), 1,1′-(5,14-dioxo-4,6,13,15-tetraazaoctadecane-1,18-diyl)bis(3-(sec-butyl)-1H-imidazol-3-ium)bis((trifluoromethyl)sulfonyl) imide and synthesized it in pure form using the mentioned methodology (vide infra) to use it as an electrolyte additive for application in LIBs. Central to our design philosophy is the use of the alkyl spacer chains between two cationic terminal groups that generally helps lowering the melting point^49^ resulting in better adaptability and lesser unwanted side reactions. Now we discuss about the rational for the choice of the anionic part of the ionic liquid. The lithium salt LiPF_6_, which is most commonly used in the conventional electrolyte system because of its proper balance in ionic conductance and wide electrochemical stability window^50^ but at higher temperature it has tendency to decompose to give PF_5_ which promotes ring opening reactions of cyclic carbonates leading to continuous electrolyte degradation^51^. NTf_2_ anion in IL helps in reducing the viscosity of the ionic liquid to make it appropriate as electrolyte. It is also well known to exhibit advantageously high resistivity towards hydrolysis^52^.

In addition to these, the IL enhances the thermal stability due to its negligible vapour pressure and adds extra safety feature to the lithium ion battery. The density functional theory (DFT) calculations provide the clue for the extra stability of the electrolyte after addition of IL. We propose the above mentioned many-fold actions as basis for designing our IL as an electrolyte additive for enhanced performance and safety of LIB.

A promising approach is demonstrated in this study by using ILs as a nominal additive to a conventional electrolyte consisting of ethylene carbonate and dimethyl carbonate (EC + DMC), leading to beneficial synergistic effects on the physicochemical properties of the resulting mixtures. This approach has several techno-commercial benefits: (i) it enhances the cyclability of the cells, (ii) it also improves long term capacity retention (iii) significant cost escalation can be avoided because of the nominal use of newly developed material and, (iv) the battery fabrication process will remain similar to the existing protocols. Item no (iii-iv) are very important as, at the early stage of adoption, one cannot expect process optimization at that transient phase which is typically characterized by high operational and capital expenditures. The synthetic protocol, physicochemical and electrochemical properties, the performance of IL and its role as an additive in EC + DMC electrolyte in LIBs are reported here.

The performance of a novel material intended for application in a battery can be demonstrated either by half- or full-cell configuration. A major limitation of the former approach is that, many of the findings might or might not be tenable in the full-cell configuration. Performance of the full-cell configuration is closest to the real-life usage and commercial applications. We, therefore, particularly demonstrate the advantages of our new IL electrolyte additive in full-cell configuration.

## Results and Discussions

**Scheme 1:**
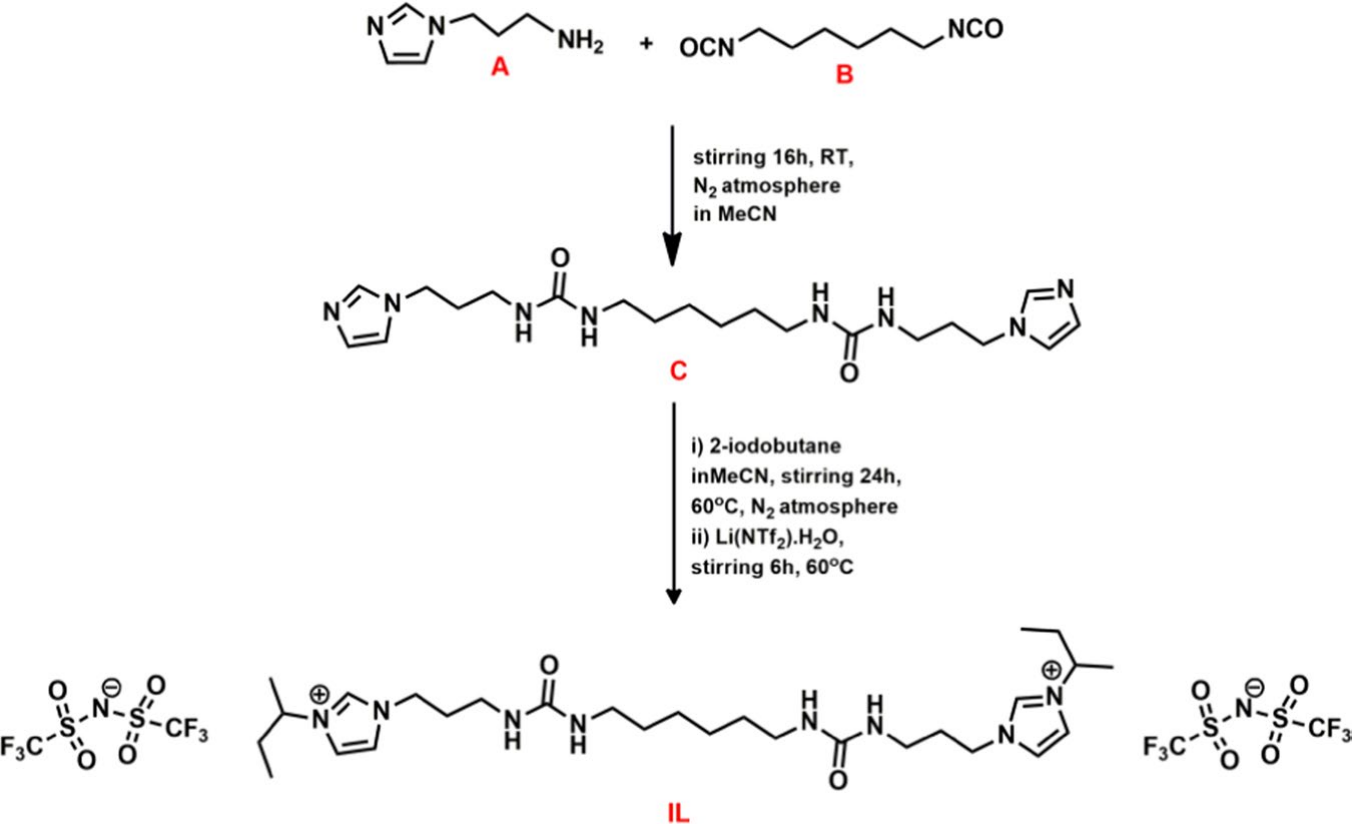
Synthetic scheme of urea functionalized dicationic ionic liquid. A, B, C denote 3-(1*H*-imidazol-1-yl)-1-propanamine, Hexamethylene diisocyanate and intermediate complex respectively (see text for detailed protocol).

The synthetic route of IL is schematically presented in Scheme-1(Materials and Methods section). In order to confirm the purity of synthesized IL, ^1^H NMR, ^13^C NMR and IR spectroscopy, ESI-mass spectrometry and elemental analyses were carried out. NMR analyses have been presented in δ scale and the chemical shift (δ) in ppm is with respect to TMS. ^1^H NMR (400 MHz, DMSOd_6_, ppm) δ = 0.77 (t, *J* = 8 Hz, 6H), 1.24 (t, *J* = 8 Hz, 4H), 1.36 (t, *J* = 8 Hz, 4H), 1.47 (d, *J* = 8 Hz, 6H), 1.81 (t, *J* = 8 Hz,4H), 1.92 (t, *J* = 8 Hz, 4 H), 2.99 (m, *J* = 8 Hz, 8H), 4.15 (t, *J* = 8 Hz, 4H), 4.40 (q, *J* = 8 Hz, 2 H), 5.90 (m, *J* = 8 Hz, 4H), 7.84 (s, 2H), 7.88 (s, 2H), 9.24 (s, 2H) (Fig. S1). ^13^C NMR (100 MHz, DMSOd6, ppm): 158.7, 135.8, 123.2, 121.3 (q, *JC-F* = 196 Hz), 118.4, 58.3, 47.3, 36.4, 31.1, 30.4, 29.5, 26.6, 20.5, 10.3 (Fig. S2). FTIR spectra was recorded for the neat IL sample and the important functional groups were identified. IR (neat, ʋ_max_/cm^−1^): 3416, (N-H), 2864 (C-H), 1647 (C=O), 1352, 1138 (Aliphatic C-N) (Fig. S3). The identity of IL was also confirmed by ESI mass spectrometry. ESI-MS m/z: calcd for C_24_H_44_N_8_O_2_ ([IL-(2NTf_2_)-(butyl) + H]^2+^), 238.18; found, 238.18; calcd for C_28_H_52_N_8_O_2_ ([IL-(2NTf_2_)]^2+^), 266.21; found, 266.20; calcd for C_26_H_44_F_6_N_9_O_6_S_2_ ([IL-(NTf_2_)-(butyl) + H]^+^), 756.28; found, 756.28; calcd for C_30_H_52_F_6_N_9_O_6_S_2_ ([IL-(NTf_2_)]^+^), 812.34; found, 812.33; calcd for C_32_H_53_F_12_N_10_O_10_S_4_ ([IL-2(NTf_2_)] +H)^+^, 1093.26; found, 1093.28 (Fig. S4). The purity of the bulk sample was further confirmed by elemental analyses of IL. Anal. calcd for C_32_H_52_F_12_N_10_O_10_S_4_: C, 35.16; H, 4.80; N, 12.81; O, 14.64; S, 11.73%. Found: C, 34.94; H, 4.84; N, 12.64; O, 14.76; S, 11.56%. Purity 99.37%.

### Physicochemical properties

In LIBs the ionic conductivity of the electrolyte plays a pivotal role. The electrical resistivity of the electrolyte solution significantly contributes to the internal resistance of the electrochemical system. So, we checked the ionic conductivity of the conventional electrolyte in presence and absence of IL at room temperature. It has been observed that after the addition of ionic liquid (20 mM) to conventional electrolyte, [1 M LiPF_6_ + (EC + DMC, 1:1)] the ionic conductivity slightly decreases from 12.20 mS cm^−1^ to 11.87 mS cm^−1^. These ionic conductivity values lie in the range of mS cm^−1^ which is in agreement with the reported ionic conductivity values for the used electrolytes for LIBs^53–55^. Moreover, electrochemical impedance spectroscopy data (vide infra) suggests that this nominal decrease in the ionic conductivity actually increases the electrode resistance which helps the formation of stable SEI in first cycle.

**Figure 1:**
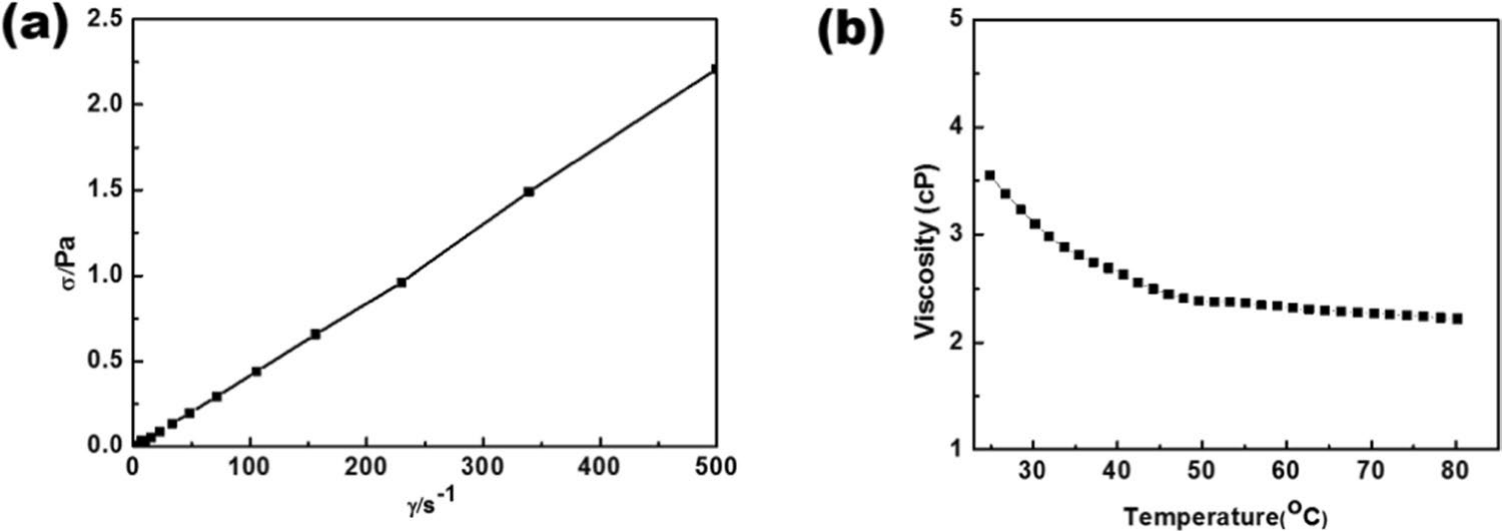
(**a**) Flow curve for equimolar mixture of EC/DMC containing 1 M LiPF_6_ + IL. (**b)** Variation of viscosities of equimolar mixture of EC/DMC containing 1 M LiPF_6_ + IL.

Further, the viscosity of an electrolyte plays an important role since it might significantly alter the transport properties. Because of strong interionic interaction, pure ionic liquid generally shows higher viscosity than commonly used organic electrolytes^56^. The viscosity of the electrolyte with IL has been measured by a steady-state flow mode. The linear evolution of shear stress vs shear rate (Fig. 1a) shows typical Newtonian behaviour. It is observed that viscosity remains almost constant with the time of shearing (Fig. S5). When the temperature was increased the viscosity of the electrolyte decreased monotonically (Fig. 1b).

**Figure 2:**
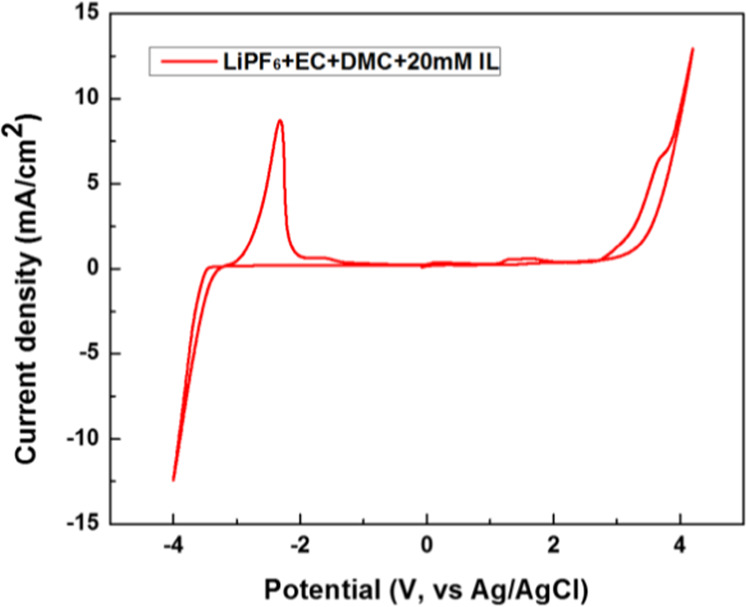
Cyclic voltammogram of [LiPF_6_ (1 M) + EC-DMC (1:1) + 20 mM IL] at Pt working electrode. Potential shown here are vs. Ag/AgCl reference electrode.

Cyclic voltammogram (CV) of electrolyte with IL additive (Fig. 2) was recorded at 300 K with a scanning rate 1 mV s^−1^ to find the electrochemical window. Determining the electrolyte stability is a non-trivial task. It has a complex relationship between the redox potentials of the solvent, reactions with other solvent molecules and electrolyte salts and moreover the surface characteristics of electrode material^57^.The oxidation peak is observed at +3.25 V and the reduction is observed at −3.40 V, both values are indicated against (E°) Ag/AgCl. These values corresponds to −0.151 V to +6.499 V vs E° (Li/Li^+^). In between this range, the electrolyte acts as an ideally polarizable solvent^58^. This is one of the desirable properties of an electrolyte in a cell, which is satisfied by this IL to be used as electrolyte additive in lithium ion battery^59–61^. Interestingly when the scan is reversed, oxidative decomposition is observed at −2.32 V Ag/AgCl. This might be the reason that this electrolyte additive works as a protective SEI layer for the electrode.

**Figure 3:**
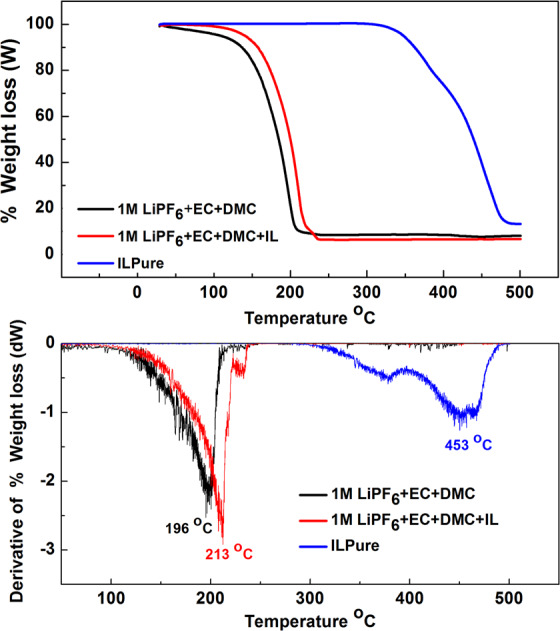
TGA curves of LiPF_6_ + EC + DMC (black; conventional electrolyte) LiPF_6_ + EC + DMC + IL (red; conventional electrolyte with nominal IL mixture proposed in this study), and Pure IL (blue; the IL designed and synthesized in this study).

The thermal properties of the electrolyte play significant role in battery safety^62^. Thermogravimetric Analysis (TGA) of (LiPF_6_ + EC + DMC), (LiPF_6_+EC + DMC + IL) and pure IL are shown in Fig. 3.

It shows the one stage decomposition behaviour. The onset of degradation temperature (Figs. S6–S8) and decomposition temperature of conventional electrolyte, electrolyte + IL and pure IL are shown in Fig. 3. It can be seen that there is essentially no weight loss for pure IL at 300 °C and less than 3% loss at 350 °C which indicates that the IL is thermally stable and releases very little volatile species. It clearly shows that by adding the IL to the organic electrolyte the thermal stability is improved (red line) and % weight loss has been reduced.

To ensure the stability at low temperature, IL was kept in −20 °C and −35 °C fridge one by one for 6 hrs. It remained liquid at those temperatures. To find out the low temperature behaviour, DSC measurement was carried out (Fig. S9). It was found that at approx. −42 °C phase change occurred. No melting transition was observed. The ionic liquid only showed the feature of a glass transition^63^. From the derivative plot it was observed that the glass transition temperature was −42.17 °C.

**Figure 4:**
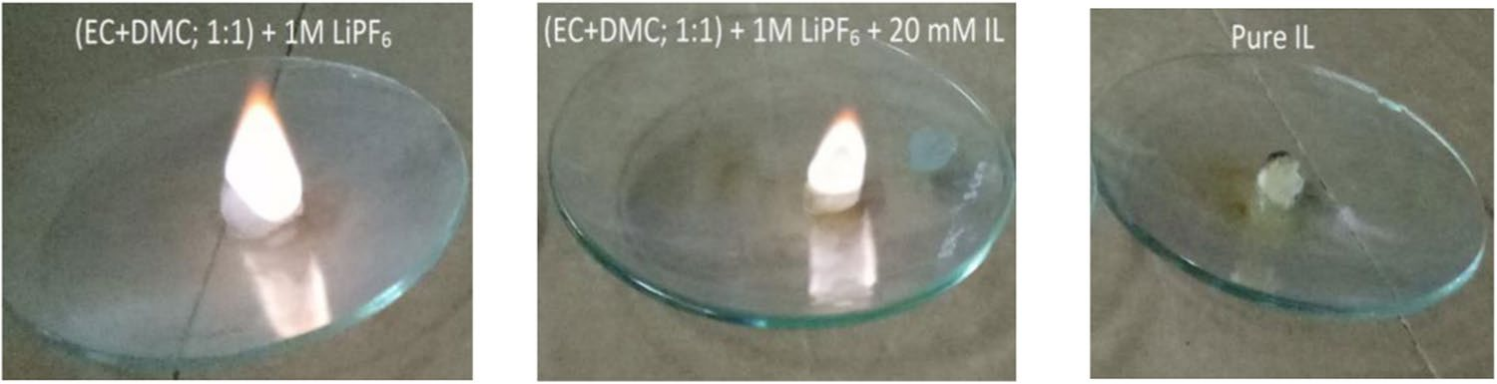
Comparative flammability test for conventional electrolyte (left), Conventional electrolyte with IL (middle) and pure IL (right).

The non-flammability, which is an intrinsic property of IL based electrolytes, also is an important criteria for the safety of LIB. Comparative flammability test for conventional electrolyte, conventional electrolyte with IL and pure IL was carried out (Fig. 4). It was observed that conventional electrolyte immediately catches fire, electrolyte with 20 mM IL slowly catches fire after15 sec and pure IL did not catch fire to produce a flame at all. Thus, it clearly demonstrates that electrolyte with IL additive is safer than the conventional organic electrolyte.

In order to have a better insight about the electrochemical window and stability of organic carbonate electrolytes and electrolyte-additive towards redox process, DFT calculation was performed using Gaussian 09 package^64^. These theoretical calculations were performed at RB3LYP level using 6–311 + G (d) basis set. One can easily correlate the HOMO and LUMO energies with molecular properties e.g. ionization potential, electronegativity and electron affinity in a semi quantitative manner^65^. Energies of the HOMO and LUMO for the optimized structures are summarized in Table 1. Here, the Hartree constant of 27.21 for unit conversion was used to get final HOMO and LUMO energy values. The obtained HOMO and LUMO energy values for EC are in accordance with the reported literature value^66,67^. According to frontier molecular orbital theory, decrease in HOMO energy stabilizes the system towards oxidation i.e. its oxidation become difficult and decrease in LUMO makes the system easily reducible. DFT calculation revealed that the HOMO energy level of IL is lower than that of the both conventional organic carbonate electrolytes EC and DMC, and hence IL is more difficult to get oxidized and thereby providing the wide electrochemical window towards oxidation. Moreover, IL has a lower energy level of LUMO than that of the organic solvents/electrolytes, indicating that IL is a better electron acceptor and hence provides weaker resistance against reduction but more likely it is capable to produce better film formation at higher potential^20^. This film modifies the structure and solid/electrolyte film and subsequently improves LIB cycle performance which is reflected in the further studies by scanning electron microscope (SEM) imaging (Fig. 8) and electrochemical impedance spectroscopy (Fig. 9).

**Table 1:** Energies of the frontier orbitals of the organic solvent electrolytes and ionic liquid as electrolyte additive.

Electrolyte	HOMO (eV)	LUMO (eV)
EC	−8.4666	−0.2772
DMC	−8.1823	−0.0666
IL	−10.0092	−5.4480

### Lithium cell performance

**Figure 5:**
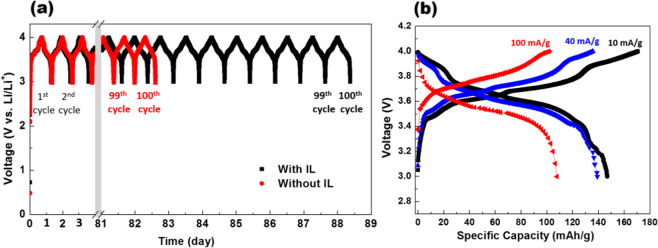
(**a**) 100 cycles of Galvanostatic (constant current) charging and discharging of the cells with IL (black colour) and without IL (red colour) as a function of time. The charge and discharge cycles were interrupted when the cell voltage exceeded 4.0 V, or dropped below 3.0 V respectively. Notice the break plot from 3 to 81 days. The cell with IL (black) outperformed the cell without IL (red) by providing at least 6 days more service. (**b**) Rate performance of the cell for electrolyte with IL at different current rate of 10 mA/g, 40 mA/g and 100 mA/g.

Figure 5a shows the results for both types of coin cells, without IL (EC + DMC) and with IL additive (EC + DMC + IL), which were galvanically charged-discharged at a current rate of at 10 mA/g for 100 cycles. It clearly demonstrated that the cells with IL additive outperformed the cells without it by providing at-least 6 days more service for the same number (100) of cycles. Before investigating different aspects of this performance enhancement, we checked the performance of cells with IL additive in different current-loading conditions because this would reveal important traits of lithiation and de-lithiation behaviour of the cells.

The cells were operated at current rate ranging from a lower value of 10 mA/g to a relatively higher value of 100 mA/g, as shown in Fig. 5b. At an intermediate current rate of 40 mA/g, the cell had nearly similar performance to the lower current rate (10 mA/g). This establishes good cell’s performance, even in more demanding situations. It is indicative of the fact that the performance boost because of IL addition (as established in Fig. 5(a) does not come at the cost of any problematic kinetic compromise. At current rate 100 mA/g, which is nearly ten times faster, the cell still retained around 73% of capacity.

**Figure 6:**
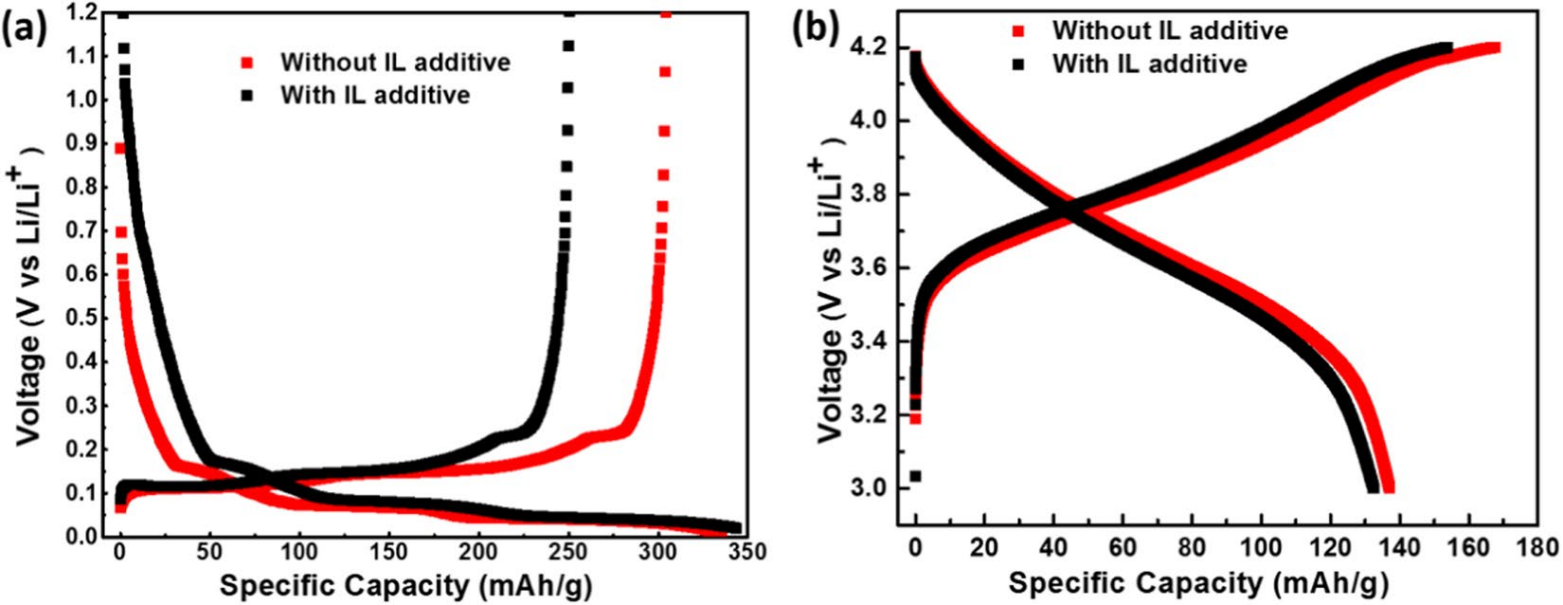
(**a**) First cycle galvanostatic (constant current at 10 mA/g) discharge-charge profile of the graphite anode half cells with IL (black colour) and without IL (red colour); (**b**). Charge-discharge profile of the NMC cathode in half cell configuration with IL (black colour) and without IL (red colour).

In order to obtain further insight of the LIBs cells with IL additive, the half-cell for individual graphite and NMC electrode were fabricated using lithium metal foil as a counter electrode. The half-cell performance of the graphite and NMC electrode was evaluated at a current rate of 10 mA/g as shown in Fig. 6(a,b) respectively. It is interesting to note that the performance of graphite anode (charge capacity) with IL additive is lower than the without IL. Though at a first glance it might seem that using of IL additive is counterproductive since it is degrading the cell performance, however, the detailed analysis presented in the following section will establish that it is actually improving the overall cell performance over a longer duration. The NMC cathode with IL and without IL shows similar capacity (charge-discharge) performance, therefore it can be attributed to the degradation of electrolyte on graphite electrode due to the lower energy level of LUMO in case of IL additive than that of the conventional electrolyte (Table 1).

**Figure 7:**
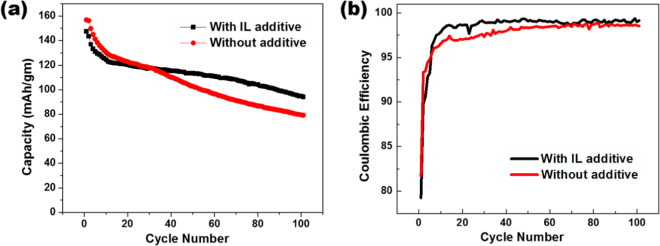
(**a**) Cyclic discharge performances of the cell in electrolyte without (red) and with IL (black); (**b**) Cyclic Coulombic efficiency performances of the cell in electrolyte without (red) and with IL (black).

**Figure 8:**
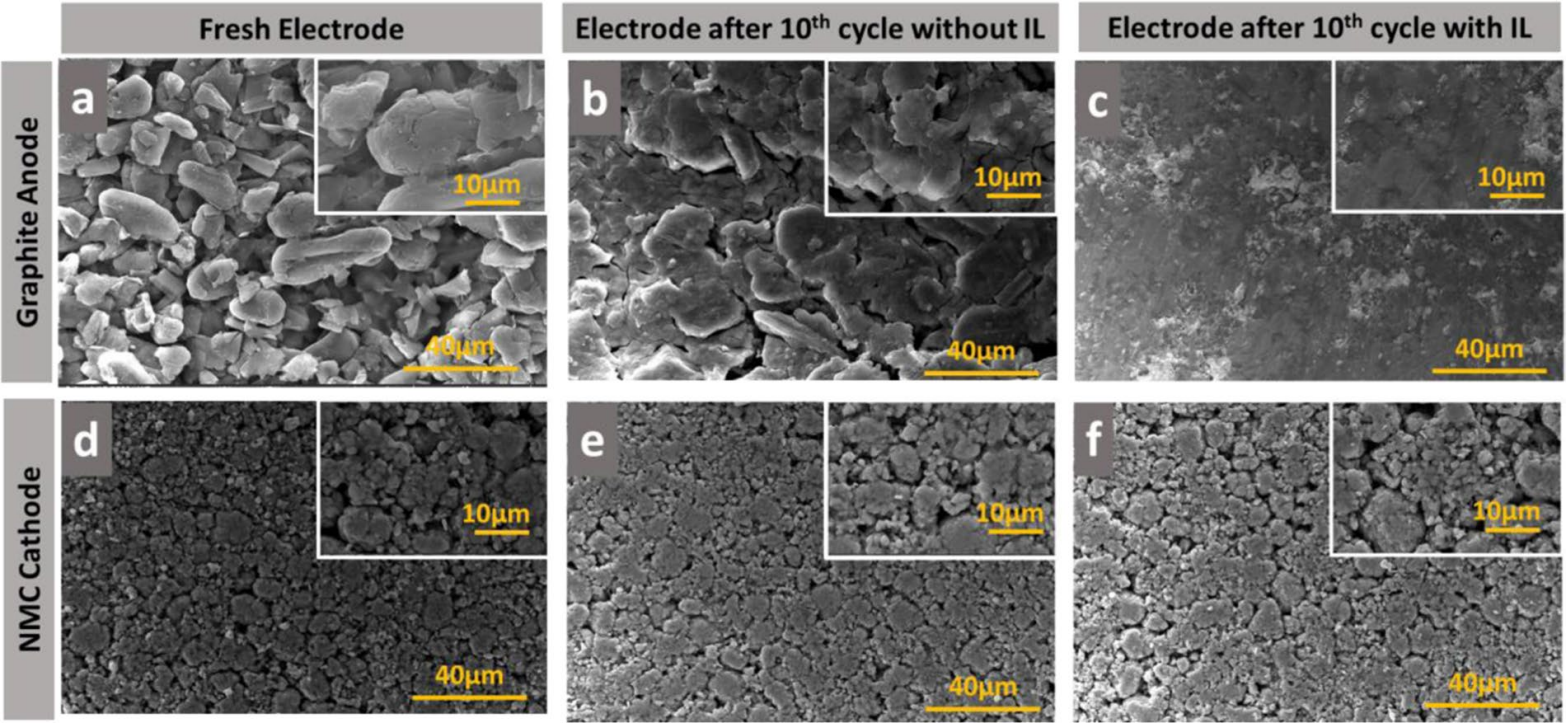
SEM microstructure of (**a)**. Graphite coated on Cu current collector before 1 st cycle; (**b)**. Graphite anode in full cell configuration without IL additive after 10^th^ cycle; (**c)**. Graphite anode in full cell configuration with IL additive after 10^th^ cycle; (**d)**. NMC coated on Al current collector before 1st cycle; (**e)**. NMC cathode in full cell configuration without IL additive after 10^th^ cycle; (**f)**. NMC cathode in full cell configuration with IL additive after 10^th^ cycle.

The cyclability of the cells with- and without-IL additive were investigated further and the results are displayed in Fig. 7a. During the first few cycles, the discharge capacity of the cell without the additive was slightly higher than the cell with the IL additive. After around 30 cycles, the trend in capacity fading for the two types of cells changed and the cell with IL additive showed better capacity than the cell without IL additive for long run. This is indicative of the fact that the addition of IL additive in electrolyte makes the mixture electrochemically more favorable in the long run inside the LIB which turns out to be beneficial for the overall cell life. If we take a much closer look, then we observe that the capacity fading for both the cells have roughly similar trend until 10 cycles, however, after elapse of 10 cycles, there is a change in slope for these two curves. This is indicative of the fact that the capacity fading for the cells with IL additive was mostly arrested after about 10 cycles (as indicated by a flatter plateau) and further investigated later (Fig. 8). It can also be observed that cells with IL additive have higher coulombic efficiency than those without IL additive as depicted in Fig. 7b. The improved coulombic efficiency, on the top of increased long-term capacity is remarkable and it is indicative of the fact that the addition of IL additive significantly slows down the decomposition of electrolyte during cycling.

Evidences for the SEI formation phenomenon at the interface of electrode and electrolyte can be obtained from the understanding of the microstructural and surface chemistry changes of the electrodes as a function of time when it is exposed to the electrolyte mixture. Therefore, scanning electron microscope (SEM) imaging and energy-dispersive X-ray spectroscopy (EDX) analysis were performed on the graphite anode and NMC cathode of the full cells both in freshly made condition as well as after completion of 10^th^ charge-discharge cycles. The fresh graphite anode and NMC cathode prior to any type of cell cycling are shown in Fig. 8a,d, respectively. The graphite anode without IL additive can be seen to be covered by the incipient non-uniform surface films (Fig. 8b) in contrast to the with IL additive, which can be seen to have a homogeneous and smooth morphology; Fig. (8c). Also, the passivation films formed over the graphite electrode with IL additive is oxygen rich when compared to the without IL additive (See Fig. S11), which is likely to be due to the formation of stable SEI over the graphite electrode with IL additive.

The cathode NMC with IL additive and without additive after cycling shows the similar morphology with the fresh NMC cathode as shown in Fig. 8(d–f). Also, the elemental distribution in the both the case has a similar composition, this implies that the addition of IL additive is not significantly affecting the surface of the NMC cathode (see Fig. S12). These results correlate well to the charge discharge profile of the graphite and NMC electrode in half cell (Fig. 6) as well energy level (HOMO-LUMO) of electrolyte and additive (Table 1).

**Figure 9:**
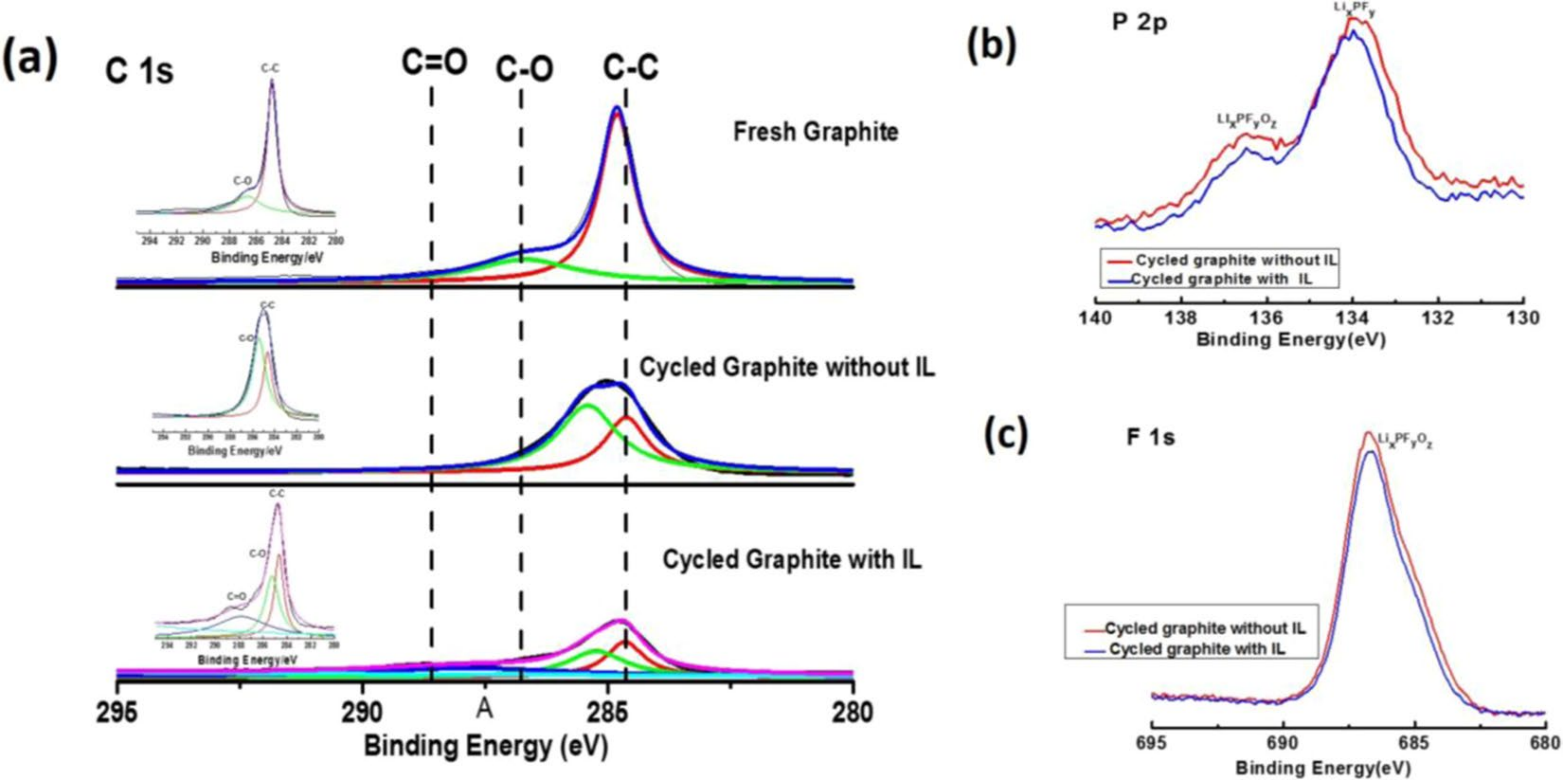
(**a**) Comparative C 1 s XPS spectra of graphite electrodes with IL and without IL after 10 cycles (inset, separate XPS graphs of fresh graphite electrodes, with and without IL additives has been given). (**b**) P 2p XPS spectra of graphite electrodes with IL and without IL after 10 cycles. (**c**) F 1 s XPS spectra of graphite electrodes with IL and without IL after 10 cycles.

**Figure 10:**
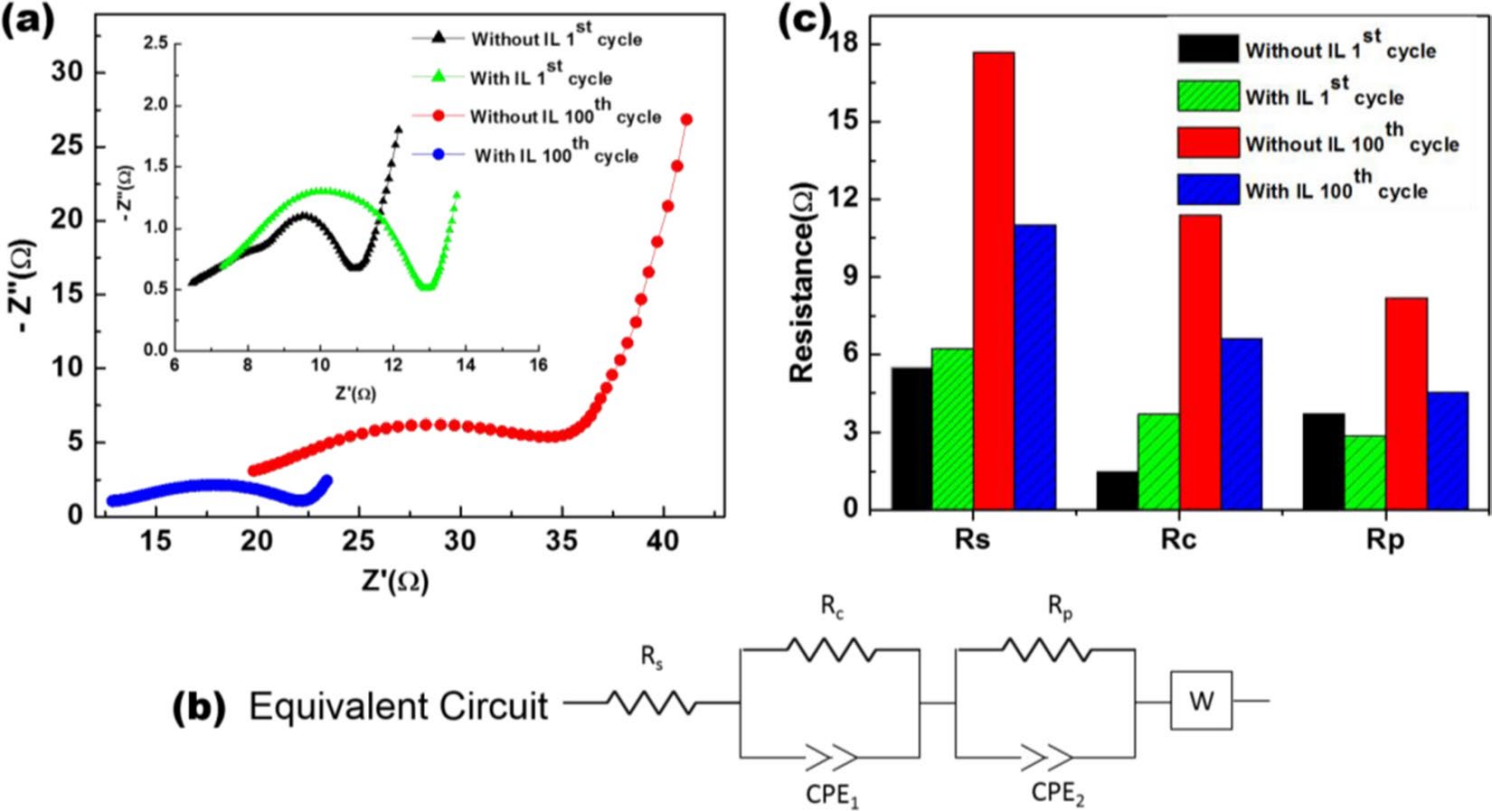
(**a**) AC impedance response of the cell for electrolyte without and with IL additive. The measurements were conducted after the 1^st^ and 100^th^ cycle of the cell. (**b**) Equivalent circuit of a lithium ion battery. (**c**) The corresponding resistance value are represented in the bar chart for the fitted equivalent circuit.

X-ray photoelectron spectroscopy (XPS) analysis of graphite electrode after 10 cycles also supports stable SEI formation. In C 1s spectra (Fig. 9a), the peak at 284.6 eV correspond to the C-C bond which is related to the carbon black (conductive agent)^68^. The peaks at 288.6 eV correspond to the C=O and at 286.6 eV correspond to the C-O of lithium carbonate on the electrode surfaces, which may be due to the oxidative decomposition of the carbonate electrolyte used^69^. The C-C peak with IL additive is significantly reduced while the C=O peak is increased with IL additive which clearly indicate that the graphite electrode surface is better covered by the passivation film in presence of IL. In P 2p spectra (Fig. 9b), two peaks, one for Li_x_PF_y_ (P-F) at 133.9 eV and another for Li_x_PF_y_O_z_ (F-P-O) at 136.4 eV can be clearly seen for both without and with IL additive. Li_x_PF_y_ and Li_x_PF_y_O_z_ are the decomposition products of LiPF_6_ salt. The intensities of these two peaks are decreased for the electrodes cycled with IL additive. This is because, the decomposition of the IL-based electrolyte generates less F-P-O and P-F intermediates than those of electrolyte without IL additive upon cycling. Therefore, we can tell that IL additive significantly decreases the amount of LiPF_6_ decomposition products to make stable SEI film at the electrode surface. F 1 s spectra (Fig. 9c) also shows the similar trend. The intensity of P-F peaks at 686.7 eV is reduced in case of IL additive. The above results indicates that the SEI layer on graphite electrode with IL additive has lower inorganic components (EDS data support the same, Fig. S11) which corresponds to the low interface impedance (Fig. 10) of the full cell after cycling. Therefore we believe that the addition of IL additive improves the SEI layer on the graphite electrode.

Further insight into it is obtained by electrochemical impedance spectroscopy (EIS) of the cells. These measurements were performed after 1^st^ and 100^th^ cycles over a frequency range from 0.1 Hz to 100 kHz measured at the corresponding open circuit potentials (OCP) with a perturbation amplitude of 10 mV. The results are presented as Nyquist plots in Fig. 10a. The impedance results were fitted to an equivalent circuit of a lithium ion battery as shown in Fig. 10b. It is comprised of R_s_, R_C_, and R_p_ representing the resistance of the Li^+^ transport through the electrolyte, SEI and electrode respectively. CPE_1_ and CPE_2_ represent the capacitance of surface film and double layer respectively^70–72^. It is important to note here that the electrolyte resistance, R_s_, is primarily governed by viscosity whereas, for film resistance R_c_, viscosity does not play any major role. For the first cycle, both R_s_ and R_c_ for the cells with IL additive are marginally higher than the without IL additive. This, therefore explains lower capacities at initial cycles for the cells with IL additive compared to the cells without it.

It is remarkable that the electrode resistance with IL is lower than that of without it even for the first cycle. This is so because the addition of IL provides a protective coating on the electrode surface which leads to the formation of stable SEI from the first cycle itself. It is well known that the electrolyte degrades with each progressive cycle, which generally manifests in the form of capacity-fading. After elapse of 100 cycles, the remnant capacity and coulombic efficiency both were higher for the cells with IL additive (Fig. 7a,b). It can be correlated to the lower values of all the three resistances (R_s_, R_c_ and R_p_) at that point (100 cycles) in Fig. 10c. Therefore, it is reasonable to conclude that the cell with IL additive showed lower decomposition of electrolyte, formation of stable SEI which is also confirmed by theoretical calculation and better electrochemical compatibility of the electrolyte mixture and electrode surface indicated by lower values of R_s_, R_c_ and R_p_ respectively after 100 cycles of operations.

## Conclusions

Using a bottom-up approach, we have designed a novel dicationic ionic liquid to be used as an additive to the conventional (EC + DMC) electrolyte for LIBs and its complete protocol for synthesis is provided. The chemical identity and purity of this IL has been confirmed by various spectroscopic techniques like Nuclear Magnetic Resonance (^1^H NMR and ^13^C NMR), Infrared Spectroscopy (IR), Electrospray Ionization-Mass Spectrometry (ESI-MS) and elemental analyses. The physicochemical properties such as ionic conductivity and temperature dependent viscosity, flammability, TGA, DSC and CV of the electrolyte have been investigated. We demonstrated significant improvements in capacity with nominal increase in coulombic efficiency after 100 cycles of operation in full-cell configuration, therefore representing near real-life performance. It is noteworthy that the cell with IL additive outperformed the cell without it by providing at-least 6 days more service for the same number (100) of cycles. After 100 cycles of operations, we also showed significantly lower resistances for Li^+^ transport through all the three media viz. electrolyte, SEI and electrode in presence of IL additive. This is indicative of sustained good health of the battery after the elapse of many cycles and enhances intrinsic safety. This is supported by the morphological studies indicating the formation of relatively stable SEI layer (when compared to the cells without IL additive) that provides performance enhancement of the LIB. We reasoned that adaption of using this additive will not have any adverse economic effect since it is advocated to be used as a nominal additive to the most widely used conventional organic electrolyte.

## Materials and Methods

### Source material

All solvents (analytical grade and spectroscopic grade) were obtained from Finar (India) and Spectrochem (India) and solvents were purified using standard literature methods. EC (99%), DMC (99%) and deuterated solvents for NMR were bought from Sigma-Aldrich (India) and used as received. 3-(1*H*-imidazol-1-yl)−1-propanamine, hexamethylene diisocyanate, 2-iodo butane, polyvinylidene fluoride (binding material), N-methyl-2-pyrrolidone (NMP solvent) and Li(NTf_2_) were procured from Sigma-Aldrich (India) and used as received. Acetylene black (conducting material) was bought from MTI Corporation.

### Characterization techniques

^1^H NMR spectra were recorded on a Bruker AVANCE 400 NMR spectrometer. The infrared spectra were recorded on a BRUKER ALPHA-T FT-IR spectrometer in the range 400–4000 cm^−1^. Electrospray ionization mass spectra (ESI-MS) of the ligands were recorded on a Bruker microTOF-Q II mass spectrometer. Elemental analysis was performed using an Elementar vario MICRO cube CHN analyzer. Karl Fischer Titration was done using Metrohm 870 KF Titrino plus. The sample was poured in titration vessel having dried methanol and result was reported in percentage (%). Viscosity measurements were conducted using an Anton Par rheometer (MCR 301). Ionic conductivity was measured with Metler Toledo combined pH and conductometer (Eutech PC 2700) at room temperature. Cyclic voltammograms were recorded on CH instruments electrochemical analyser (Model 620D) with three electrode system (Pt working electrode, Pt wire as auxiliary electrode and Ag/AgCl reference electrode). Thermogravimetric analysis (TGA) was carried out by Netzsch STA 449 F5 under argon atmosphere from room temperature to 500 °C with a heating rate 10 °C/min. Low temperature differential scanning calorimetry (DSC) was performed by PerkinElmer DSC 8000 under nitrogen atmosphere from −75 °C to 80 °C with a cooling/heating rate 10 °C/min. The flammability tests were performed by taking three cotton plugs (1 cm diameter and 0.07 g weight each) on a watch glass soaked with i) conventional electrolyte, ii) conventional electrolyte added with 20 mM IL and iii) pure IL. All three cotton plugs were exposed to the gas lighter’s flame and their ignition time was monitored. The coin cells were assembled in an argon filled glove box (M. Braun, Germany) with H_2_O < 0.5 ppm and O_2_ < 0.5 ppm. Neware BTS-5V10 mA battery tester was employed for the cycling and rate capability measurement of the cells. Electrochemical impedance spectroscopy (EIS) of the cells were performed by an AUTOLAB PGSTAT302N (Metrohm). The quantum chemical calculations were carried out using Gaussian 09 program^73^. The ground state structures have been optimized with density functional theory (DFT) at RB3LYP level using 6–311 + G (d) basis set. SEM has been carried out in FEI Apreo LoVac and EDX analysis in Oxford Instruments. XPS has been performed using a Thermofisher Scientific K-Alpha instrument.

### Synthesis of 1,1′-(5,14-dioxo-4,6,13,15-tetraazaoctadecane-1,18-diyl)bis(3-(sec-butyl)-1H-imidazol-3-ium) bis((trifluoromethyl)sulfonyl)imide (IL)

A flask containing a magnetic stirring bar was charged with the solution of 3-(1H-imidazol-1-yl)-1-propanamine, A (1.625 g, 13 mmol) in 20 mL acetonitrile and to it hexamethylene diisocyanate, B (1.0 g, 5.9 mmol) was added drop-wise under inert (N_2_) atmosphere, Scheme 1. The reaction mixture was stirred overnight at room temperature. Then the solvent was removed under reduced pressure to remove the excess volatile amine. The resulting residue, C was dried overnight in vacuo. The solid residue was then redissolved in acetonitrile under inert atmosphere and (2.3 g, 12.50 mmol) of 2-iodo butane was added to it. The reaction mixture was then stirred with gentle heating (60 °C) for 24 hrs. The solvent was removed under reduced pressure leaving a sticky white solid. The resulting solid residue was dissolved in water and the aqueous solution was washed with ether. To the aqueous solution, was added 1.2 equivalent solution of Li(NTf_2_) in 100 mL of water. The mixture was stirred overnight at 60 °C, which resulted in a biphasic system comprised of an upper aqueous layer and a lower product phase. The aqueous phase was decanted and the product was washed with water (4 ×100 mL) to remove excess Li(NTf_2_). Water was removed by the azeotropic distillation of the compound in acetonitrile-toluene mixture and further solvent was removed under reduced pressure. Finally, the trace amount of water was removed to the best possible extent by drying the product in lyophilizer overnight. To ensure that the IL is moisture free, Karl Fischer Titration was performed and it was observed that IL has 0% water present in it (Fig. S10). Yield: 75%.

### Electrochemical cell fabrication

In most automotive LIBs, lithium nickel cobalt manganese oxide Li(Ni*x*Co*y*Mnz)O_2_ (NMC) is employed as the cathode active material^74,75^ due to its high specific capacity, potential with respect to Li*/*Li^+^ and environment friendly character. Here the Layered Lithium Nickel Manganese Cobalt Oxide (NMC111) cathode material is used with composition 33.33% Ni, 33.33% Mn, and 33.33% Co (LiNi_1/3_Mn_1/3_Co_1/3_O_2_). For nearly all commercially available cells, graphite is used as the anode active material^76^. Therefore, NMC111 cathode and graphite anode are the natural choices for fabrication of our LIBs. To prepare the slurry for the cathode, NMC111, acetylene black, and Polyvinylidene Fluoride (PVdF solvent) were mixed at a weight ratio of 8:1:1 in N-methylpyrrolidinone. The slurry was coated onto an aluminum current collector using doctor blading (MTI corporation) having 13.9 mg/cm^2^ active mass loading of NMC, the electrode plate was subsequently dried at 120 °C under vacuum. The process was same for anode, where NMC was replaced by graphite and the slurry was coated onto a copper foil, having active mass loading of 6.5 mg/cm^2^. During the calendering process for the graphite anode, which was coated onto the current collector foil, the flat flake shaped graphite particles got oriented parallel to the foil. Since this direction was perpendicular to the electrode-electrode orientation, it increased tortuosity anisotropy which therefore forced the Lithium ions to traverse along greater diffusion distance^77^.

Using graphite and NMC111 electrodes, 2032-type coin cells were fabricated by assembling a glassy filter as a separator, the commercial electrolytes containing EC and DMC in a ratio of 1:1 (v/v) with 1 M lithium hexaflourophosphate (LiPF_6_) salt. For fabricating cells with IL additives, 20 mM IL added into commercial electrolyte while all other protocols remained identical.

## Supplementary information

Supplementary Information.
