# Supplementary material for: Synthesis, characterization and application of a non-flammable dicationic ionic liquid in lithium-ion battery as electrolyte additive
Source: Sci Rep. 2020 Jun 15;10:9606. doi: 10.1038/s41598-020-66341-x (PMC7295740; doi:10.1038/s41598-020-66341-x)
Supplement: Supplementary file 1 — Supplementary Information. [file 41598_2020_66341_MOESM1_ESM.pdf]

Supporting information

Synthesis, characterization and application of a non-flammable dicationic ionic liquid in lithium-ion battery as electrolyte additive

Kajari Chatterjee [a], Anil D. Pathak [a,c], Avinash Lakma [b], Chandra Shekhar Sharma[c], Kisor Kumar Sahu [a]* and Akhilesh Kumar Singh [b]*

[a]School of Minerals, Metallurgical and Materials Engineering, Indian Institute of Technology Bhubaneswar, Bhubaneswar, 752050, India.

[b]School of Basic Sciences, Indian Institute of Technology Bhubaneswar, Bhubaneswar, 752050, India.

[c]Creative & Advanced Research Based On Nanomaterials (CARBON) Laboratory, Department of Chemical Engineering, Indian Institute of Technology, Kandi, Hyderabad 502285, Telangana, India.

*Corresponding Authors:

E-mail address: aksingh@iitbbs.ac.in; Tel: +91-674-7135114

E-mail address: kisorsahu@iitbbs.ac.in; Tel: +91-674-7136920.

Index:

S. No.	Supporting information details
**Fig. S1.**	^{1}H NMR (400 MHz) spectra for IL[1] in DMSO-d_6.
**Fig. S2.**	^{13}C NMR (100 MHz) spectra for IL[1] in DMSO-d_6.
**Fig. S3.**	FTIR spectra of IL.
**Fig. S4.**	ESI-MS Spectra for IL.
**Fig. S5.**	Viscosity Curve of (EC+DMC+LiPF6) + 20 mM IL
**Fig. S6.**	TGA graph of EC+DMC+$LiPF_6$ as a function of temperature
**Fig. S7.**	TGA graph of EC+DMC+$LiPF_6$+IL as a function of temperature
**Fig. S8.**	TGA graph of pure IL as a function of temperature
**Fig. S9.**	Low temperature DSC graph of pure IL as a function of temperature
**Fig. S10.**	Water content measurement for IL
**Fig. S11.**	EDS elemental analysis for graphite anode before and after 10th cycling
**Fig. S12.**	EDS elemental analysis for NMC cathode before and after 10th cycling

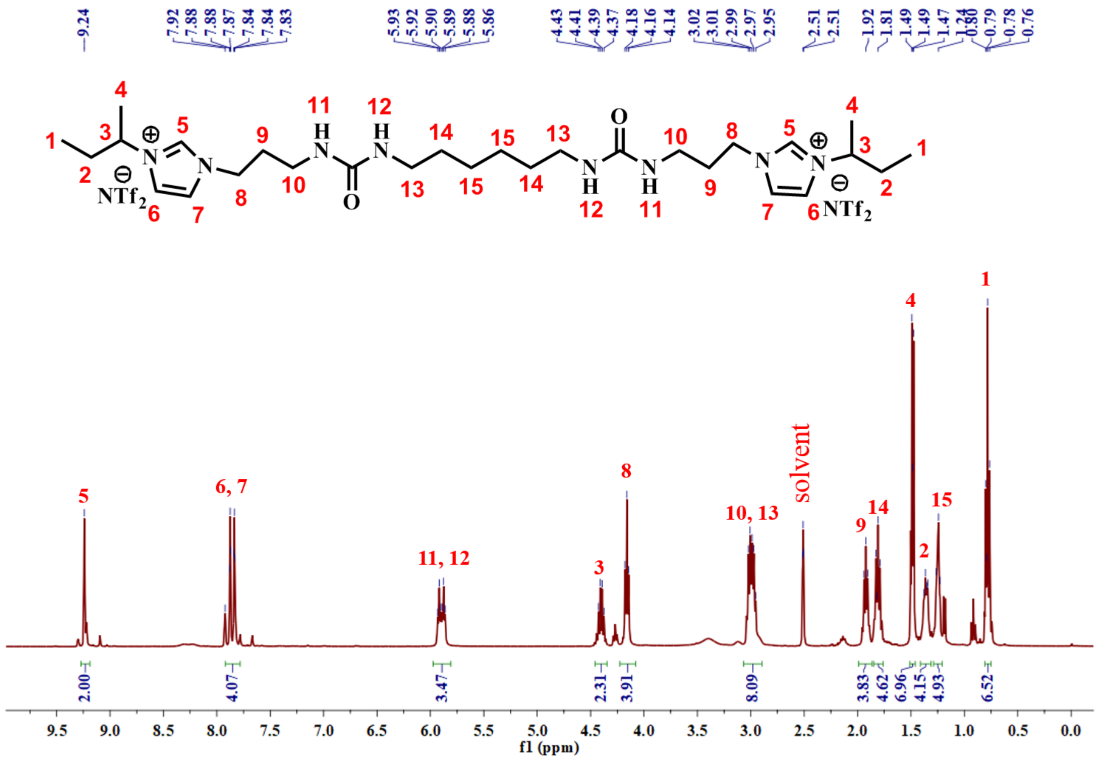

Fig. S1. ^{1}H NMR (400 MHz) spectra for IL[1] in DMSO-d_6.

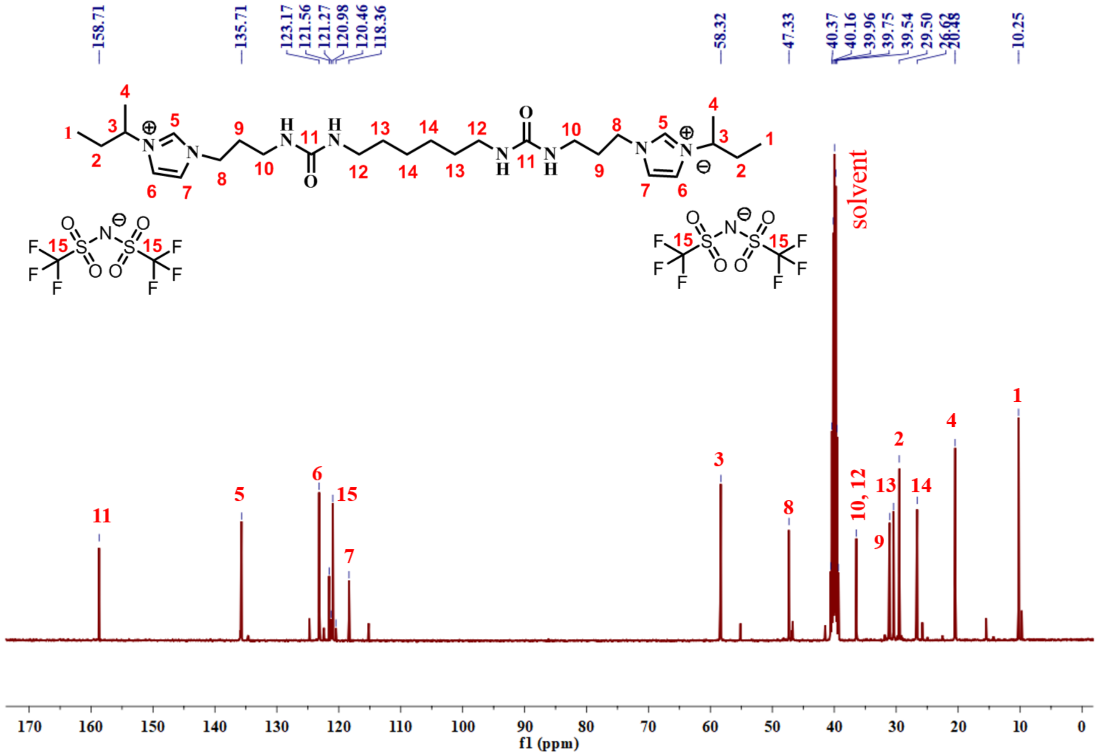

Fig. S2. ^{13}C NMR (100 MHz) spectra for IL[1] in DMSO-d_6.

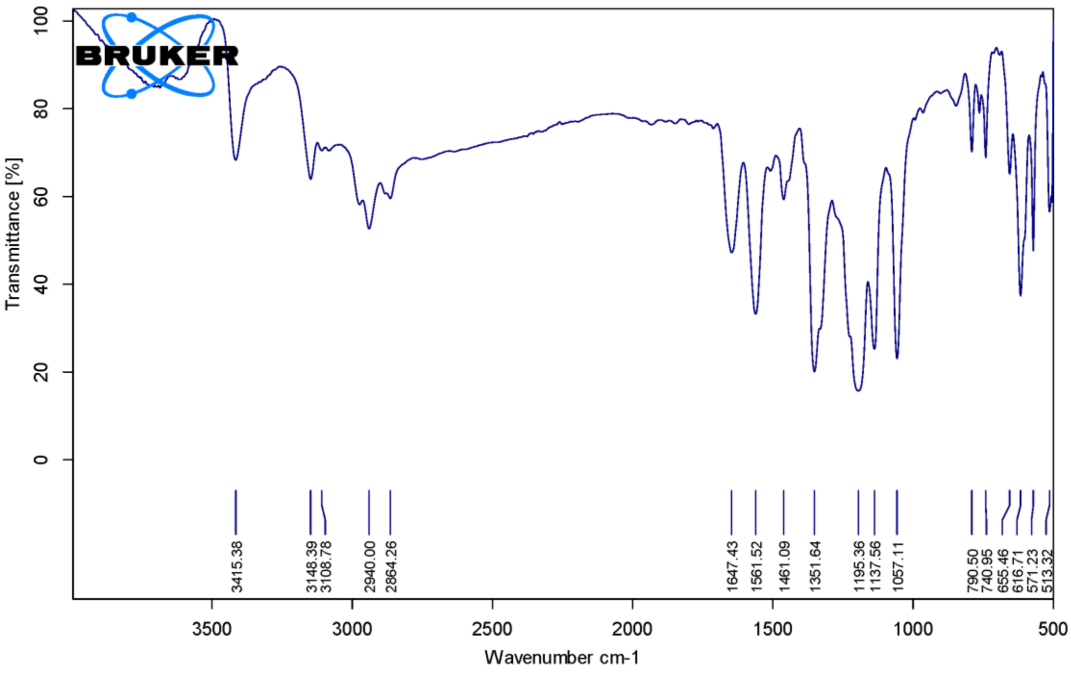

Fig. S3. FTIR spectra of IL.

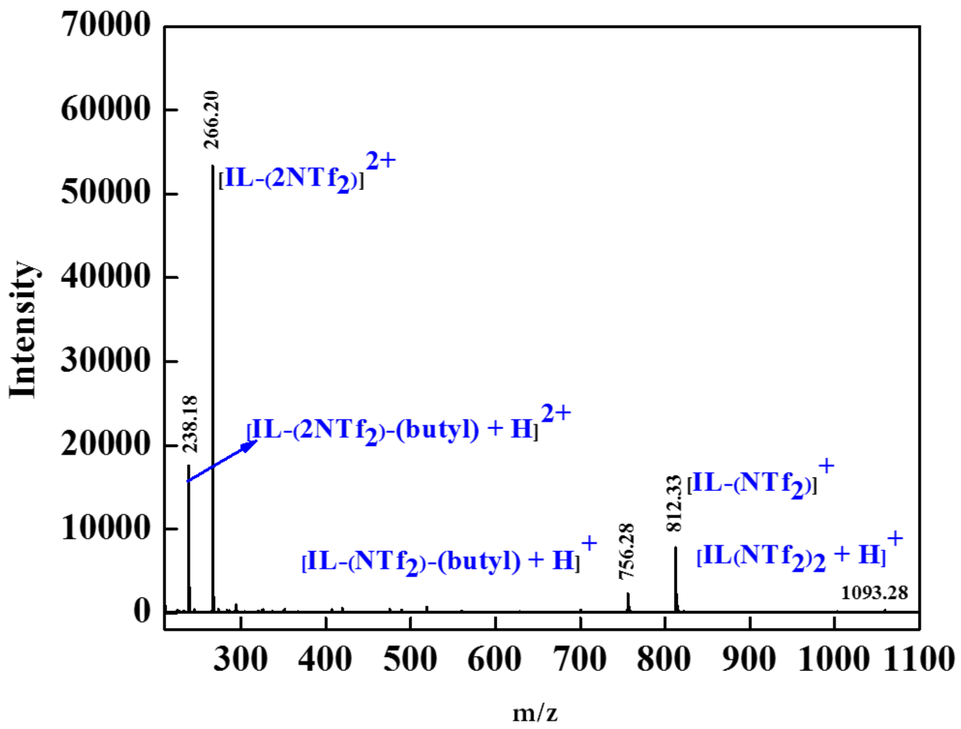

Fig. S4. ESI-MS Spectra of IL.

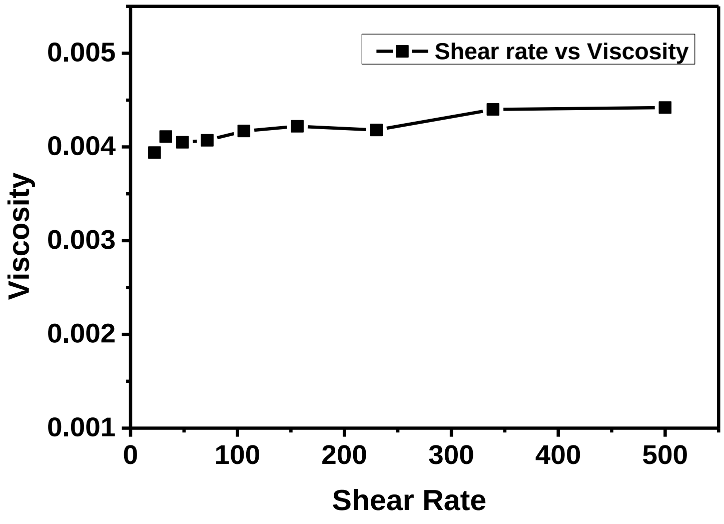

Fig. S5. Viscosity of $LiPF_6$ + (EC+DMC) + IL as a function of shear rate.

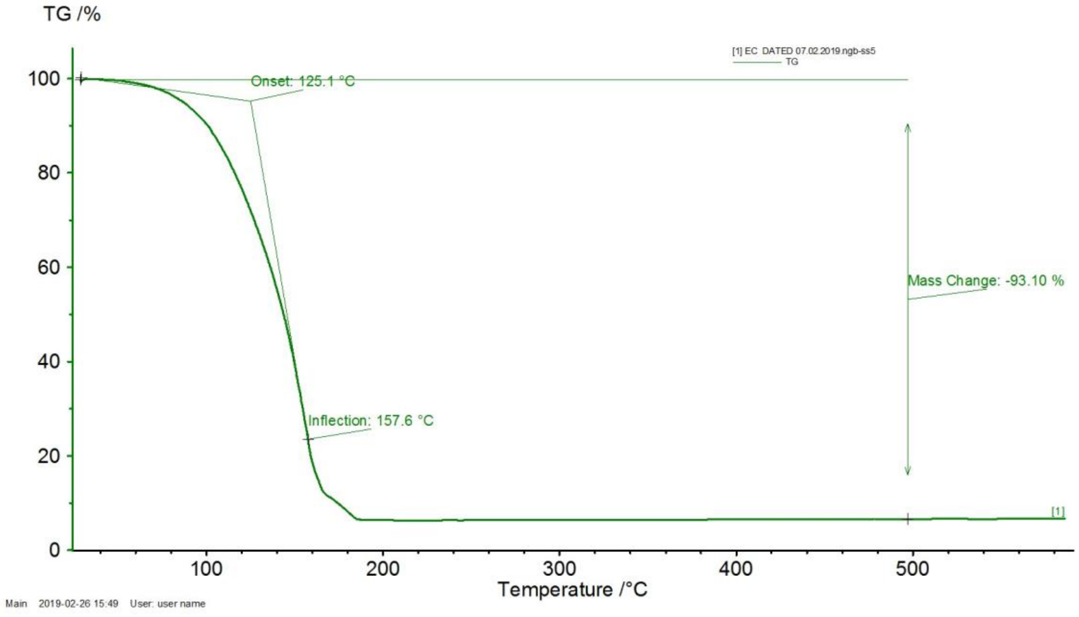

Fig. S6. TGA graph of EC+DMC+$LiPF_6$ as a function of temperature.

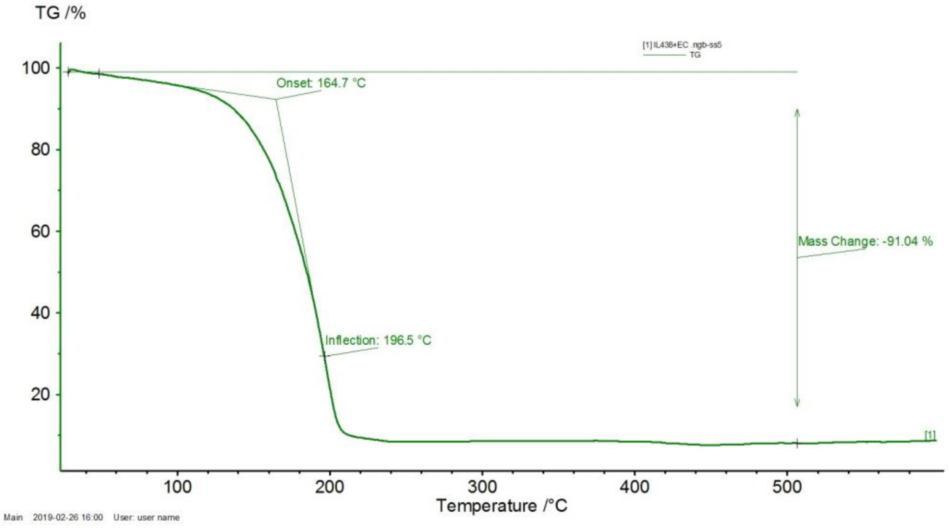

Fig. S7. TGA graph of EC+DMC+$LiPF_6$+IL as a function of temperature.

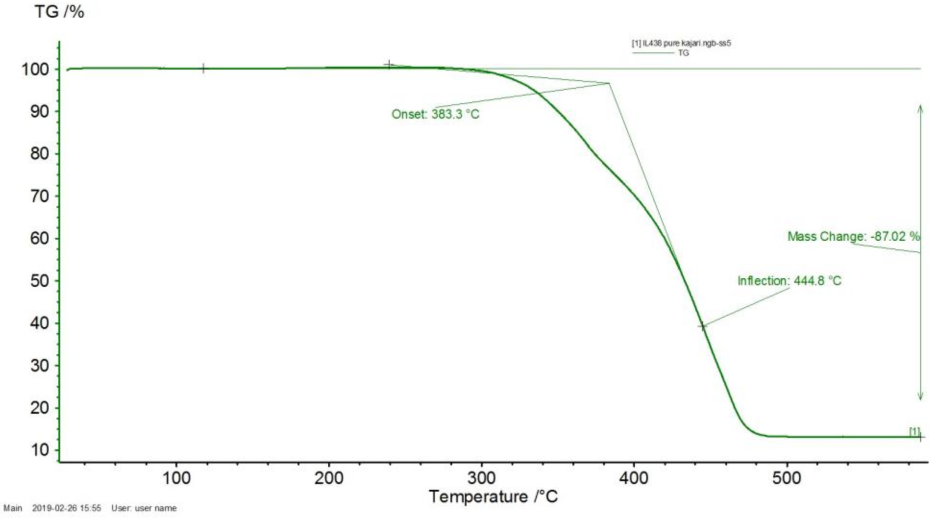

Fig. S8. TGA graph of pure IL as a function of temperature.

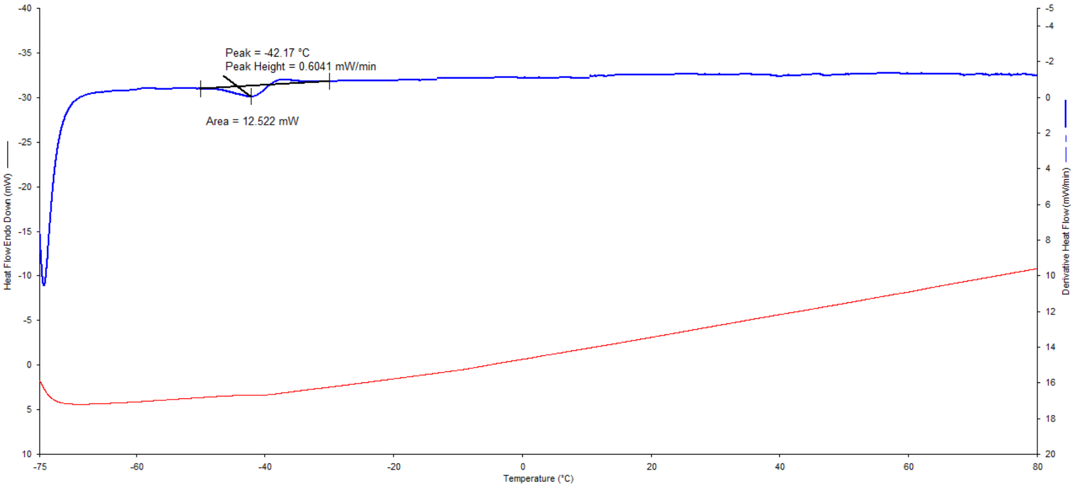

Fig. S9. Low temperature DSC graph of pure IL as a function of temperature.

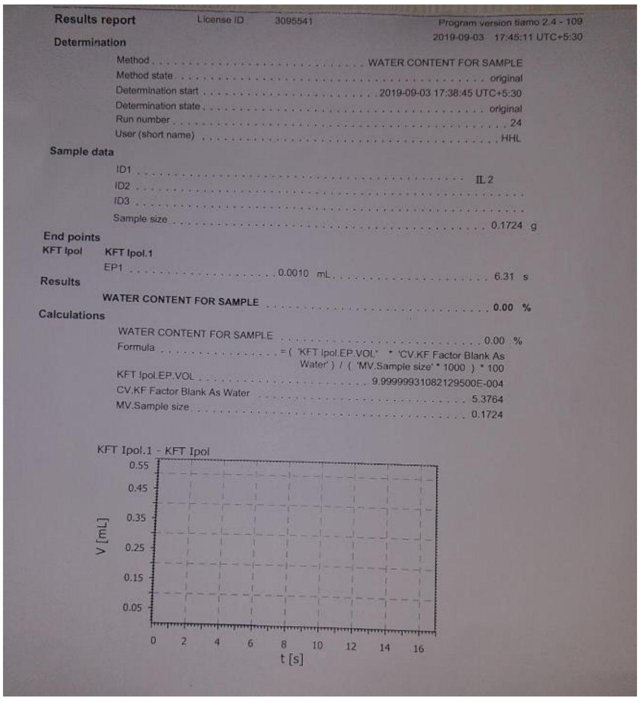

Results report License ID 3095541 Program version tiamo 2.4 - 109

2019-09-03 17:45:11 UTC+5:30

Determination

Method . . . . WATER CONTENT FOR SAMPLE
Method state . . . . original
Determination start . . . . 2019-09-03 17:38:45 UTC+5:30
Determination state . . . . original
Run number 24
User (short name) . . . . HHL

Sample data

ID1 . . . . IL 2
ID2 . . . .
ID3 . . . .
Sample size . . . . 0.1724 g

End points

KFT Ipol **KFT Ipol.1**
EP1 . . . . 0.0010 mL . . . . 6.31 s

Results

WATER CONTENT FOR SAMPLE . . . . 0.00 %

Calculations

WATER CONTENT FOR SAMPLE . . . . 0.00 %
Formula . . . . = ('KFT Ipol.EP.VOL' * 'CV.KF Factor Blank As Water') / ('MV.Sample size' * 1000) * 100
KFT Ipol.EP.VOL 9.99999931082129500E-004
CV.KF Factor Blank As Water 5.3764
MV.Sample size 0.1724

KFT Ipol.1 - KFT Ipol
V [mL]
0.55
0.45
0.35
0.25
0.15
0.05
0 2 4 6 8 10 12 14 16
t [s]

Fig. S10. Water content measurement for IL.

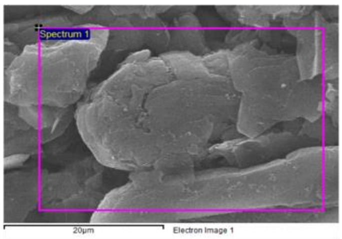

**Bare Graphite
Before cycling**

Element	Weight%	Atomic%
C K	92.17	99.14
Cu K	2.12	0.43
Pd L	0.94	0.11
Au M	4.77	0.31
Totals	100.00	

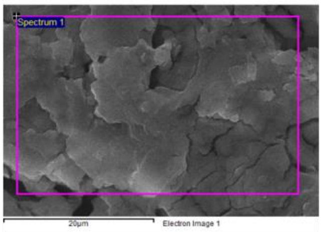

**Graphite electrode without
Additive In full cell after 10 cycle**

Element	Weight%	Atomic%
C K	45.39	64.32
O K	8.03	8.54
F K	22.79	20.42
P K	5.31	2.92
S K	0.12	0.07
Cu K	11.47	3.07
Pd L	0.95	0.15
Au M	5.94	0.51
Totals	100.00	

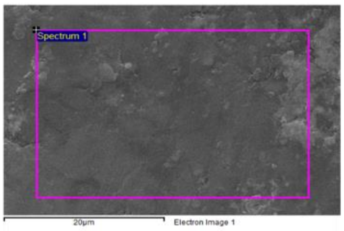

**Graphite electrode with IL additive
In full cell after 10 cycle**

Element	Weight%	Atomic%
C K	23.56	43.94
O K	32.15	45.03
F K	1.71	2.02
P K	1.32	0.95
S K	0.26	0.18
Cu K	11.14	3.93
Pd L	5.67	1.19
Au M	24.19	2.75
Totals	100.00	

Fig. S11. EDS elemental analysis for graphite anode before and after 10th cycling

Bare NMC

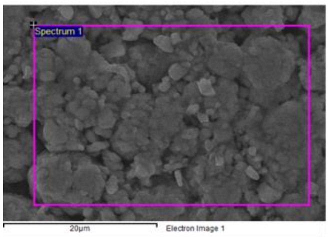

Element	Weight%	Atomic%
C K	16.40	33.36
O K	24.13	36.87
Mn K	17.49	9.45
Co K	16.71	8.69
Ni K	17.07	8.86
Pd L	1.09	0.25
Au M	6.56	0.81
Totals	100.00	

**NMC_Full cell after 10 cycle
Without IL additive**

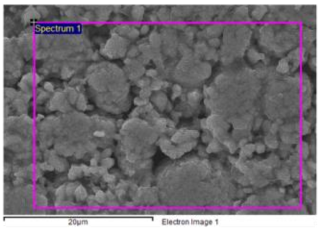

Element	Weight%	Atomic%
C K	15.02	29.80
N K	-1.12	-1.90
O K	23.70	35.30
F K	13.61	17.07
Al K	1.01	0.89
P K	1.24	0.96
S K	0.05	0.03
Mn K	13.55	5.88
Co K	13.65	5.52
Ni K	14.15	5.74
Pd L	0.88	0.20
Au M	4.26	0.52
Totals	100.00	

**NMC electrode in Full cell
after 10 cycle with IL addditive**

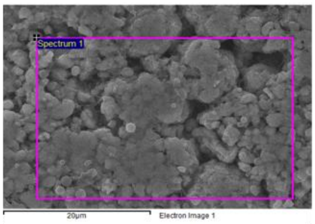

Element	Weight%	Atomic%
C K	13.95	27.95
N K	-1.12	-1.93
O K	23.29	35.02
F K	15.13	19.16
Al K	1.16	1.03
P K	1.35	1.05
S K	0.05	0.04
Mn K	13.36	5.85
Co K	12.89	5.26
Ni K	14.04	5.76
Pd L	0.82	0.18
Au M	5.07	0.62
Totals	100.00	

Fig. S12. EDS elemental analysis for NMC cathode before and after 10th cycling
